# Workload Influence on Fatigue Related Psychological and Physiological Performance Changes of Aviators

**DOI:** 10.1371/journal.pone.0087121

**Published:** 2014-02-05

**Authors:** Jin Ma, Ru-Meng Ma, Xi-Wen Liu, Ka Bian, Zhi-Hong Wen, Xiao-Jing Li, Zuo-Ming Zhang, Wen-Dong Hu

**Affiliations:** 1 Department of Aerospace Medical Equipment, Faculty of Aerospace Medicine, Fourth Military Medical University. No 169, Xi’an, China; 2 Department of Clinical Aerospace Medicine, Faculty of Aerospace Medicine, Fourth Military Medical University. No 169, Xi’an, China; 3 Department of experimental surgery, Tangdu Hospital, Fourth Military Medical University. No 1, Xi’an, China; 4 Department of Basic Nursing, Nursing School, Fourth Military Medical University. No 169, Xi’an, China; 5 Department of Otolaryngology-Head and Neck Surgery, Tangdu Hospital, Fourth Military Medical University. No 1, Xi’an, China; Universidad Europea de Madrid, Spain

## Abstract

**Objective:**

We evaluated a variety of non-invasive physiological technologies and a series of test approaches for examination of aviator performances under conditions of mental workload in order to provide a standard real-time test for physiological and psychological pilot fatigue assessments.

**Methods:**

Twenty-one male aviators were selected for a simulated flight in a hypobaric cabin with artificial altitude conditions of 2400 meter above sea level. The simulated flight lasted for 1.5 h, and was repeated for two times with an intervening 0.5 h rest period outside the hypobaric cabin. Subjective criteria (a fatigue assessment instrument [FAI]) and objective criteria (a standing-position balance test as well as a critical flicker fusion frequency (CFF) test) were used for fatigue evaluations.

**Results:**

No significant change was observed in the FAI scores before and after the simulated flight, indicating that there was no subjective fatigue feeling among the participants. However, significant differences were observed in the standing-position balance and CFF tests among the subjects, suggesting that psychophysiological indexes can reflect mental changes caused by workload to a certain extent. The CFF test was the simplest and clearly indicated the occurrence of workload influences on pilot performances after a simulated flight.

**Conclusions:**

Results showed that the CFF test was the easiest way to detect workload caused mental changes after a simulated flight in a hypobaric cabin and reflected the psychophysiological state of aviators. We suggest that this test might be used as an effective routine method for evaluating the workload influences on mental conditions of aviators.

## Introduction

Aviators are prone to experience physical and mental fatigue due to cabin noise, vibration, pressure changes, long flight durations, irregular working times and lack of sleep caused by above reasons, while fatigue seriously can affect flight operations of pilots. According to a previous report, fatigue of aviators is a major reason of flight accidents [1]. The common effects of fatigue on aviators are decreased visual acuity, mind numbing, single-mindedness, unresponsiveness, judgment errors, memory loss, unstable organization, indifference and irascibility. The severity of aviators fatigue symptoms are also depending on the flight altitude, which can be divided into three levels: symptomless level (an altitude below 3000 m), compensatory level (3000–5000 m) and dangerous level (above 7000 m). At the compensatory level, the heart rate and pulmonary ventilation increases, indicating that the compensatory response of the human body develops some hypoxia adaption mechanisms at this altitude and symptoms are not serious if the period is short or the condition is in a stationary state, but even at the compensatory level mental functions and work abilities of aviators may already be affected [2]. At the dangerous level, the oxygen demand of the body cannot be satisfied by compensatory responses and mental and motor coordination dysfunctions occur after exposure to this area in a short time. However, modern aircrafts generally are equipped with an automatic pressure regulation and in most airplanes the cabin atmospheric pressure can be adjusted to a pressure equivalent of 1500 to 2000 meters while the flight altitude is 9000 to 10000 meters above the sea level and a simulated flight in a hypobaric cabin with a 2400 m altitude atmospheric pressure is a close to reality model for studying mental workload of aviators.

There are various evaluating methods for flight caused fatigue and basically, they include subjective and objective tests. The fatigue assessment instrument (FAI) is a kind of subjective fatigue evaluation questionnaire, which has been designed by Schwartz et al. in 1993 [3]. One of the objective evaluating methods is the CFF test, which has been used for psychological and behavioral evaluation with a normal critical value of 25 Hz–55 Hz [4]. Studies suggested, that the CFF decreases by 0.5 Hz–6 Hz after highly focusing, relatively overuse of the eyes and during dull, repetitive work. The balance testing method is a physiological index, which relies on the central nervous system capacity of the aviator to regulate visual, proprioceptive and vestibular sensations and control the movement effectors. Although an evaluation to which degree aviators are under mental workload is of great significance, no explicit evaluation criteria on aviators fatigue have been established in China at present, whereas comprehensive measures were used to decrease and prevent mental workload caused fatigues of aviators including the following methods: (1) a nap lasting for 20 min–2 h could nearly compensate sleep insufficiency and recover the pilot; (2) to prevent jet lags, sleep delay when the plane is heading west and get up or sleep ahead of time when heading east; (3) maintenance of a regular diet with high protein content during day time and high carbohydrate containing food, fruits and dairy products at night; (4) alleviation of work pressure, keep the brain in good condition and improve the awakening by proper physical exercise when flying at night. All of these measures were recommended to prevent but could not timely detected aviator’s fatigues and thus could not completely avoid flight accidents caused by fatigue. In this study, a hypobaric cabin with a simulated altitude of 2400 m was used to simulate the mental workload of a pilot, which could mimic a high altitude flight without serious hypoxia. The physiological and psychological indexes of the participants in the simulated flight were monitored and changes of those indexes that were related with and accurately reflected flight fatigue were determined, thereby providing the basis for significant clinical data ensuring safe flights.

## Methods

### Study subjects

Twenty-one healthy male junior college students (from 20 to 22 years, height of 175.4±3.1 cm and weight of 66.6±5.1 kg) were selected for this study from March 21, 2010 to March 25, 2010 with four subjects tested each day. All of them were right-handed, led a regular life, without smoking, excessive drinking with a regular sleeping time from 22:00 to 6:00 without sleep disorders and no neurological and psychiatric or medication history. The research was approved by the Ethical committee of the Fourth Military Medical University and all participants provided their written informed consent to participate in this study.

### Methods and procedures

The simulated flight schedule was from 8:30 am to 3:00 pm and the participants were required to have adequate normal rest for two days before the test. In addition, they were required not to consume tea, tobacco, alcohol, coffee and other central nervous system (CNS) stimulants or depressants a day before the test to be emotionally and psychologically stable. The subjects were instructed to enter the hypobaric cabin at 9:30 am for the initial stimulated 2400 m altitude flight for 1.5 h. The time necessary for all psychophysiological tests was 30 minutes and one cycle consisted of 30 minutes test, 1.5 h flight simulation and 30 minutes test again. After the first cycle, it was lunchtime for 30 min and then subjects did another cycle after which the trial was finished. The subjects were required to do certain activities such as, watching movies or playing games during the stimulated flight to keep their minds active and there were one or two persons in charge with supervision.

### Cabin altitude selection

Cabin pressure and oxygen levels decrease with increased flight altitude. Above 4000 m, the oxygen levels in the human body cannot maintain normal activities and hypoxia occurs. Cabin air pressure increases using the bleed air from the compressor of the gas turbine jet engine, whereas the piston engine is equipped with a specific supercharger for cabin pressurization to maintain cabin pressures equal to 2400–4000 m altitudes. Cabin air pressure is generally maintained and stabilized at an altitude of <3000 m to guarantee passenger’s comfort and health. Therefore, the stimulated flight altitude in the hypobaric cabin in this study was maintained at 2400 m.

### Test methods

#### (1) FAI test

The FAI comprised 29 participant statements each of them related with fatigue and had the following four factors or subscales: Factor 1: the fatigue severity scale that quantitatively measured the extent of fatigue. Factor 2: the environment-specific scale that measured the correlation of fatigue to specific situations (heat, cold, mental stress, and others) and determined whether the fatigue is context-specific. Factor 3: the outcome scale, which measured the mental effects caused by fatigue, such as reduction in patience, desire and concentration. Factor 4: the rest or sleep-responding scale that measures the effect of rest or sleep on fatigue [3].

#### (2) Balance test

**Figure 1:**
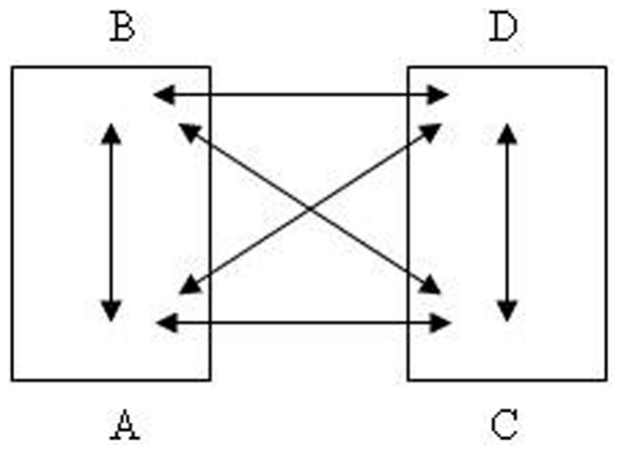
Balance pressure plate model.

A Tetrax Balance Testing Instrument manufactured in Israel was used in this study. The Tetrax balance system consists of a pressure platform with four separate plates on it. The subjects stood on the platform with each of their heels and toes on one of two plates fitting into the metal foot sketches. Vertical pressure was then transmitted to the platform, which was translated into a fluctuating signal processed later in a computer for analyzing the balance and position of the subjects. Position recording was conducted according to the following principle: Human balance and posture control is based on the coordination of pressure fluctuations generated from the four pivot points, which are the two heels and toes of each subject. The pressures generated from the four pivot points as well as the interaction among them were recorded. The measured indexes included following indexes: (A) Falling index, which was one of the balance parameters recorded as a result of falling-influencing factors and showed the risk of falling, scored as mild, medium and high. (B) Basic stability, which was determined through the measurement of all fluctuation data on the four plates and showed the overall stability of the subjects. This indicator represents the “Steady Index” (ST), which is increasing with increased instability and vice versa. (C) Weight distribution ratio and weight distribution harmony degree, which represents the percentage of weight on each of the four plates, expressed as A%, B%, C%, and D% (Fig. 1). It can be used to observe the asymmetric weight distribution between the two feet, the heel and toes of either foot, or the heels and toes of the same foot. Furthermore, diagonal differentiation was also determined. Another index was the Weight distribution index (WDI), which referred to the square of the weight distribution divided by 25% of the standard deviation. The two additional parameters of weight distribution LEFT% and HEEL% indicate the weight distributions on the left foot and on each heel. (D) The pressure mode synchronization of the heels and toes: in a normal individual, the tracking signals of the heels and toes show complementary patterns (Fig. 1), and the two sets of heels and soles show coordinative synchrony. This interaction can be expressed by the “synchrony coefficient”, ranging from −1000 (fully complementary with opposite sign waveforms between two platforms) to +1000 (completely coordinative with the same sign waveforms between two platforms). The pressure mode synchronization includes six combinations as follows: A–B, C–D, B–D, A–C, B–C, and A–D, among which A–B, C–D, B–C, and A–D were complementary, whereas B–D and A–C were coordinative.

(E) Fourier transformation of postural sway is a mathematical algorithm, which can be used for waves of any origin and shows the strength of postural sway at different frequencies. The normal position refers to the position of high strength at a low frequency of approximately 0.1 Hz, indicating that the body position is controlled by a normal vestibulo-ocular reflex. A low and medium frequency oscillation of 0.3 Hz starts when balance cannot be effectively maintained in the low-frequency oscillation. These oscillations are physiological phenomena caused by vestibular problems, drunkenness, fatigue and exhaustion. We employed eight positions in total: 1. NO (normal position, eyes open, solid surface). The subject stands straight on the test platform, looking ahead. This position is basic and serves as reference point for comparisons with other positions in this balance test. 2. NC (normal position, eyes closed, solid surface): The subject stands straight on the test platform, with closed eyes. This position reveals the impact of visual cues in terms of stability. 3. PO (opened eyes on pillows): the subjects stand on pillows with eyes opens looking ahead. The pillows lessen the sense of proprioception, which controls balance. 4. PC (closed eyes on pillows): the subjects stand on the pillows with eyes closed. The perceptions of vision, balance and position senses are limited, indicating vestibular compression. 5. HR: Head turned to the right by 45°, with eyes closed. 6. HL: head turned to the left by 45°, with eyes closed. 7. HB: head turned backwards by 30°, with eyes closed: this position is especially sensitive to disorders of the central nervous system and vestibular system. 8. HF: head bent forward about 30°, with eyes closed.

#### (3) Critical flicker fusion frequency test

An EP403 light spot scintillometer (Scientific and Educational Instrument Factory of East China Normal University) was used to determine the Critical flicker fusion frequency (CFF). Testing method: Under a room temperature of 10°C to 15°C, the subjects were instructed to be in a sitting position in a quiet environment. Following the sequence of “↑ ↓ ↓ ↑” and a frequency (intermittent red flickering light stimulus) ranging from 7 Hz to 80 Hz, the trial lasted for about 5 min and was conducted twice with eight sets of data per subject recorded by experimenters. The arithmetic mean of these data (per subject) was the CFF.

### Statistical analyses

SPSS17.0 was used for the statistical analyses. Normal distribution was examined by a Kolgomorov-Smirnov test. All continuous variables are shown as mean and standard deviation (± sd). T test was used for the comparisons between before and after the trial. A multiple-factor repetitive measurement and analysis of variance test was used for comparisons between the end of the first cycle, begin of the second cycle and pre-trial values. For comparison of two groups, a paired t test was applied. A p value <0.05 was considered significant.

## Results

### The subjective evaluation revealed no mental workload awareness of the subjects

The FAI results of the subjects before and after the simulated flight showed that no significant differences of fatigue severity factors, environment-specific factors, outcome factors, or the rest and sleep-responding factors were stated between before and after trials (Table 1) were stated, indicating that no mental workload was recognized by subjective scales.

**Table 1:** FAI Scores before and after the trial.

Factor	Before trial	After trial	T	*P*
Severity scale (factor1)	3.72±1.45	3.80±1.03	0.151	0.702
Environment-specific scale(factor2)	4.21±1.05	4.33±1.15	0.239	0.630
Outcome scale (factor 3)	4.74±1.28	4.16±1.45	1.781	0.197
Rest, sleep-responding scale(factor 4)	5.05±2.00	5.48±1.76	0.679	0.420

### The objective evaluating criteria revealed that changes of ST, weight distribution and CFF resulted from the mental workload

There was an obvious difference of basic position NO between before and after the trial, whereas NC did not significantly differ, indicating that vision increases unsteadiness. There was no significant difference between the test results in position PO, but they were significant different compared to NO (P<0.05), indicating that pillows might lessen the imbalance sense of subjects. For position PC the test result at the end of the first cycle and begin of the second cycle were significantly different compared to the pre-trial values (P = 0.003), but there was no significant difference between before and after the trial (Table 2). The stability differences in other position with head turning were not statistically significant, suggesting that steady index was only correlated with vision and consistence of the standing platform material.

**Table 2:** Stability results of the eight positions in the trial.

	(1)	(2)	(3)	(4)
Position	Before trial	After 1^st^ time out of cabin	Before 2^nd^ time into cabin	After trial
NO	12.42±2.91	14.54±3.58▴	13.75±4.55	15.45±4.70▴
NC	17.96±7.06	18.21±8.14	17.12±6.86	16.96±5.69
PO	17.72±5.97*	18.35±4.95*	18.67±4.83*	17.91±6.90*
PC	29.05±10.16*	24.63±7.84*▴	24.90±7.79*▴	26.79±10.95
HR	16.44±6.05	15.94±5.26	15.40±5.26	16.32±4.01
HL	16.42±5.78	17.12±5.26	15.89±5.20	16.66±6.26
HB	17.23±5.28*	18.09±6.87	16.89±4.56	17.02±4.94
HF	17.50±7.45	16.54±5.58	16.88±5.94	17.24±6.61

Note: *indicate that there is a statistical significance compared with position NO, *P*<0.05;

▴ indicates a significant difference between values before and after each measurement, *P*<0.05.

### Weight eistribution

The weight distribution results revealed that the differences of C% was statistically significant different in position NC (P = 0.036), while in position PO, the differences of A% (P = 0.024), B% (P = 0.029) and (P = 0.035) as well as heel % (P = 0.0240 were statistically significant different. The differences of weight distribution in other positions were not statistically significant (Table 3).

**Table 3:** Weight distribution results of eight positions in the trial.

Position	%	(1)	(2)	(3)	(4)
		Before the trial	After 1^st^ timeout of cabin	Before 2^nd^ timeinto cabin	After the trial
NO	A%	23.96±6.07	25.60±5.83	24.56±5.75	25.36±6.56
	B%	26.75±6.48	25.77±5.28	26.41±6.12	25.95±5.91
	C%	23.06±6.96	21.17±5.58	21.82±6.40	22.28±5.94
	D%	26.23±6.02	27.46±5.24	27.21±5.88	26.41±4.93
	LEFT%	50.71±3.89	51.37±4.78	50.97±3.86	51.31±3.95
	HEEL%	47.02±11.55	46.77±9.16	46.39±10.64	47.64±9.67
NC	A%	23.84±5.34	24.54±5.69	24.24±5.39	23.98±6.84
	B%	26.66±5.14	26.75±6.09	26.33±6.37	26.65±6.44
	C%	22.82±6.03	20.70±6.31▴	22.40±6.46	22.06±5.68
	D%	26.68±4.92	28.00±4.74	27.03±5.66	27.31±4.82
	LEFT%	50.49±3.85	51.29±5.18	50.58±3.74	50.63±3.87
	HEEL%	46.66±9.15	45.24±9.38	46.64±40.53	46.03±9.93
PO	A%	19.72±4.71	19.77±6.14	21.30±5.34▴	20.26±5.36
	B%	31.21±5.17	30.87±6.25▴	29.23±5.33▴	29.45±5.32▴
	C%	18.72±5.26	18.66±5.15	20.10±5.23	19.83±4.53
	D%	30.53±5.28	30.70±5.31	29.37±4.89	30.46±5.28
	LEFT%	50.94±3.28	50.46±3.37	50.53±3.51	49.71±3.29
	HEEL%	38.44±9.00	34.43±10.34	41.10±9.28▴	40.09±9.53
PC	A%	22.16±5.16	21.50±6.03	22.68±5.97	21.27±7.09
	B%	28.74±4.79	28.96±5.71	27.71±6.02	28.56±6.04
	C%	20.67±4.28	20.17±4.92	21.33±5.74	20.67±5.79
	D%	28.42±5.23	29.37±5.57	28.28±4.87	29.50±6.56
	LEFT%	50.91±3.83	50.45±4.19	50.39±4.53	49.83±3.98
	HEEL%	42.83±8.47	41.67±9.72	44.01±9.78	41.94±11.83
HR	A%	24.46±4.68	26.18±5.69	25.00±4.82	24.37±6.17
	B%	25.43±5.89	24.77±6.16	25.22±6.75	25.87±6.29
	C%	23.84±7.40	22.48±6.32	23.64±6.54	22.97±5.96
	D%	26.27±5.09	26.57±3.74	26.13±5.48	26.78±4.78
	LEFT%	49.89±4.16	50.95±5.10	50.22±3.82	50.25±3.77
	HEEL%	48.30±9.91	48.66±8.62	48.65±10.36	47.35±9.74
HL	A%	24.49±5.45	25.89±4.66	25.67±5.40	24.83±6.57
	B%	26.45±5.68	25.46±5.84	25.15±6.89	25.69±7.08
	C%	23.00±6.98	22.04±6.55	24.00±6.45	23.72±6.57
	D%	26.06±5.13	26.61±4.09	25.19±5.53	25.77±5.39
	LEFT%	50.94±4.35	51.35±4.78	50.82±4.07	50.52±3.63
	HEEL%	47.48±10.08	47.93±8.57	49.66±10.64	48.54±11.20
HB	A%	23.57±4.52	24.72±5.54	24.26±5.28	23.96±6.27
	B%	26.93±5.34	26.71±6.34	26.78±7.02	27.91±7.31
	C%	22.52±6.97	20.82±6.54	22.82±7.16	21.94±7.12
	D%	26.99±4.56	27.75±4.32	26.67±6.03	26.91±5.45
	LEFT%	50.49±4.04	51.43±5.01	51.05±3.64	51.15±3.92
	HEEL%	46.08±9.09	45.54±9.30	46.53±10.89	45.90±11.46
HF	A%	26.63±5.19	26.90±5.47	27.48±5.94	27.08±6.60
	B%	23.76±5.65	23.91±6.08	23.72±6.95	23.42±7.19
	C%	24.83±7.60	24.11±7.25	24.94±6.65	25.03±6.76
	D%	24.78±5.30	25.08±5.25	23.86±5.69	24.46±5.13
	LEFT%	50.39±4.25	50.81±4.98	51.20±3.98	50.51±3.91
	HEEL%	51.46±10.34	51.01±9.93	52.42±11.01	52.11±11.58

▴ indicates differences compared to pre-trial, p<0.05.

The WDI results also showed significant differences for NO (*P* = 0.030) as well as for PO (*P* = 0.050) and (*P* = 0.025) in (Table 4). In NO, the results of WDI were similar with that of ST, while in PO significant differences of WDI occurred between before and after the trial.

**Table 4:** Results of WDI for eight positions in the trial.

Position	Before the trial (1)	After 1^st^ time out of cabin (2)	Before 2^nd^ time into cabin (3)	After the trial (4)
NO	5.49±3.42	5.19±2.74	5.46±3.11	5.41±2.52▴
NC	4.78±2.81	5.68±2.67	5.43±2.89	5.73±2.48
PO	6.97±3.23	7.22±3.68	5.74±3.46▴	6.03±3.85▴
PC	5.59±2.44	6.06±3.32	5.58±3.03	6.32±4.01
HR	5.21±2.60	5.12±2.48	5.24±2.71	5.41±2.35
HL	5.43±2.29	5.22±1.86	5.49±2.43	5.81±2.57
HB	5.07±2.59	5.68±2.56	5.85±2.98	6.00±3.23
HF	5.34±2.71	5.38±2.81	5.88±2.47	5.94±2.57

▴ indicates differences compared to pre-trial, p<0.05.

### The fourier transformation coefficients

The Fourier transformation coefficients before and after the test showed differences for PO (P = 0.048) as well as for HL (P = 0.04) and (P = 0.009). The Fourier transform coefficients of the other positions did not show any statistically significant change (Table 5).

**Table 5:** Results of Fourier transformation coefficients of eight positions in the trial.

Position	Before the trial (1)	After 1^st^ time out of cabin (2)	Before 2^nd^ time into cabin (3)	After the trial (4)
NO	0.88±0.14	0.91±0.07	0.93±0.07	0.90±0.09
NC	0.85±0.15	0.84±0.18	0.85±0.16	0.81±0.14
PO	0.86±0.09	0.90±0.10	0.91±0.05	0.91±0.06▴
PC	0.88±0.12	0.82±0.17	0.87±0.11	0.87±0.10
HR	0.85±0.18	0.82±0.22	0.78±0.14	0.82±0.15
HL	0.92±0.07	0.84±0.15▴	0.84±0.15▴	0.85±0.15
HB	0.87±0.15	0.84±0.16	0.85±0.12	0.85±0.16
HF	0.84±0.17	0.88±0.11	0.80±0.22	0.80±0.18
Falling Index	23.90±19.19	26.00±20.44	27.81±25.27	28.86±22.62

▴ indicates differences compared to pre-trial, p<0.05.

### Critical flicker fusion frequency (CFF)

The results of CFF before and after trial showed that the overall CFF became smaller with statistical significance *P* = 0.05 (Table 6).

**Table 6:** Results of CFF determinations (±s) (Hz).

Before trial (1)	After 1^st^ time out of cabin (2)	Before 2^nd^ time into cabin (3)	After the trial (4)
39.66±3.19	38.11±3.92	38.34±2.49	37.50±2.69▴

▴ indicates a significant difference compared to pre-trial, *P*<0.05.

Taken together, we found that no mental workload caused fatigue changes were observed by the subjects before and after the trial by subjective evaluating criteria, but with the three objective evaluating criteria methods, including steady index, weight distribution and CFF, significant differences before and after the trial could be demonstrated, indicating mental workload for the subjects. In addition, the changes of CFF were the easiest to determine and might be routinely used as an effective indicator for aviator’s fatigue.

## Discussion

In the FAI scale before and after the trial, except factor 3 the other three factors showed values which were not statistical significant, indicating that no fatigue state was recognized before and after the trial, probably because of a small and inadequate sample size in the present study. However, the FAI fatigue scale itself is a subjective evaluating method, which cannot exclude the influence of subjective biases. There might have been individual differences of the participants in their perception of fatigue and their individual motivation, such as their interests, seriousness and sincerity in participating in the trial, which may have influenced the evaluation. Therefore, we consider the FAI scale as a biased index determination for the mental overload caused fatigue of aviators. Balance function, as one of the important body functions, relies on the coordinated messages from the visual, proprioceptive and vestibular senses, as well as motor control functions of the whole nervous system. Foreign studies showed that the human balance and posture control functions are affected by several factors, such as gender, age, height, and weight [5]–[8] as well as alcohol and cigarette consumption, outside noise, body fatigue and moods [9]. In the present study, the stability coefficients showed, that no significant differences occurred in each indicator before and after the flight, except for NO and PC positions. Of the eight positions, NO (normal position, eyes open, solid surface) served as basic position and was used as reference for comparison with the other positions and PO scored significantly higher than NO (*P*<0.05), indicating that the balance signal through the pillow with no direct contact to the pressure platform lessened the subjects’ balance perception, which was also visible in the weight distribution, WDI and Fourier transformation data after the fist cycle of the experiment. In the first three time point test results of the stability test, the mean PC was significantly higher than that of NO, indicating that an effect on balance could be noted when the subjects were required to stand on pillows with their eyes closed. This position limited the perception of vision and proprioception, thus leading to the control of the vestibular nerve, which is the third center of balance. Otherwise, in the tests on weight distribution, WDI, Fourier transformation coefficients and the simultaneous weight detection, besides HL changes at the second and third time point of the Fourier transformation coefficients, the results showed no statistically significant differences, indicating that the subjects’ weight distribution on the four pressure boards were stable and equal, their physical center of gravity did not change and their balance and posture control were constantly excellent without pillows. In other words, the body of the subjects had been slightly or not at all influenced in the simulated flight. The CFF is the index for the ability of human eyes to distinguish flickering light and depends on the interaction between visual stimulation and psychophysiological skills. After highly focused and relatively tight-visual and repetitive task, CFF values decreased by 0.5 Hz–6 Hz [10]. In this trial, mean CFF values decreased by 2.16 Hz (2.45%) after the test, suggesting that CFF is a relatively sensitive detection index for fatigue. The results in this study are consistent with those in the study conducted by Ge et al. [11] on the relationship of cross-zone pilot CFF values with flight time and conditions. Of all testing indexes, the change of CFF was relative obvious and the other indexes showed no or limited changes. However, our study had some drawbacks, such as the inadequate subject load in each stimulated flight and the inadequate sample size. Therefore, it will be necessary to improve the design of the study further.

### Conclusions

In this study, a simulated flight mode was used to detect mental fatigue of aviators using non-invasive physiopsychological indexes and we found that the CFF value might become a novel method and technology to evaluate the mental workload influences on fatigue of aviators, thereby laying the foundation for further studies on correlations in the future.
